# Multi-omics characterization of new and aged Daqu reveals region-specific microbial succession and metabolic signatures in Maotai-flavor liquor fermentation

**DOI:** 10.1128/spectrum.03775-25

**Published:** 2026-05-18

**Authors:** Kejia Wang, Dongya Zhang, Ke Shen, Yun Qiu, Bin Deng, Jianli Zhou, Shuyi Qiu

**Affiliations:** 1 College of Life Sciences, Guizhou University, Guiyang, Guizhou, People's Republic of China; 2 Guizhou Province Key Laboratory of Fermentation Engineering and Biopharmacy, School of Liquor and Food Engineering, Guizhou University 71206, Guiyang, Guizhou, People's Republic of China; 3 Guizhou Light Industry Polytechnic University 562658 , Guiyang, Guizhou, People's Republic of China; Chengdu University 74707, Chengdu, Sichuan, China

**Keywords:** Maotai-flavor liquor, microbiome, metagenomics, metabolomics, flavor metabolism, regional differentiation

## Abstract

Daqu is an essential fermentation starter that drives the formation of the characteristic flavor of Maotai-flavor liquor, yet the ecological and metabolic mechanisms underlying its regional differentiation and maturation remain poorly resolved. Here, we performed genome-resolved metagenomic and untargeted metabolomic analyses on 48 new and aged Daqu samples collected from four major Maotai-flavor liquor-producing regions in Guizhou Province, China. We reconstructed 163 high-quality metagenome-assembled genomes (MAGs) spanning 16 bacterial and 3 archaeal phyla and identified 2,642 metabolites across ionization modes. Distinct regional microbial signatures were observed, with Jinsha Daqu showing the greatest genomic diversity and unique MAGs, whereas Maotai Daqu exhibited the highest community similarity with other regions. Aged Daqu significantly increased microbial richness and functional capacity, enriching thermophilic and spore-forming taxa (e.g., *Bacillus*, *Lentibacillus*, *Kroppenstedtia*) and enhancing carbohydrate-active enzymes (GH13, GH43, and GH3), amino acid degradation, lipid metabolism, and secondary metabolic pathways. Metabolomic profiling revealed elevated amino acid derivatives, fatty acids, esters, and phenolic compounds in aged Daqu, indicating intensified biochemical activity. Multi-omics integration linked dominant microorganisms—including *Bacillus thuringiensis*, *Actinomycetaceae bacterium,* and *Methylocaldum szegediense* to pyrazine biosynthesis, amino acid catabolism, and lipid oxidation, forming coordinated microbial–metabolite modules that underlie region-specific flavor precursor formation. These findings establish a mechanistic model in which microbial terroir, aging-driven succession, and metabolic specialization jointly shape the maturation and flavor potential of Maotai-flavor liquor.

**IMPORTANCE:**

This study provides the first genome-resolved, multi-omics framework for understanding how geographic origin and storage aging co-regulate the ecological assembly, functional specialization, and metabolic transformation of Maotai-flavor liquor. By linking specific MAGs, functional pathways, and key flavor precursors, our results offer mechanistic insights into microbial terroir and provide a scientific foundation for microbiome-guided optimization of Maotai-flavor liquor quality.

## INTRODUCTION

Maotai-flavor liquor, one of China’s most representative traditional spirits, is renowned for its complex aroma and layered flavor profile (1). Its production relies on solid-state fermentation mediated by a starter known as Daqu, which provides both enzymatic activity and a diverse microbial community that drives flavor compound formation (2). Daqu therefore functions not merely as a fermentation substrate but as a key microbial ecosystem and biochemical hub determining the quality and stability of Baijiu fermentation.

Extensive studies have characterized the microbial and biochemical composition of Daqu, showing that bacterial and fungal communities vary with production temperature (high, medium, or low), manufacturing conditions, and geographic origin (3, 4). For example, high-temperature Daqu is enriched in *Bacillaceae*, whereas low-temperature Daqu contains more *Lactobacillales* and *Enterobacterales* (4). The growing application of the “terroir” concept to Baijiu (Chinese distilled liquor) further emphasizes the influence of local environmental microbiota, raw materials, and regional climate on microbial community assembly and function. Microbiome analyses have revealed that Daqu from different provinces harbor distinct microbial consortia, such as *Kroppenstedtia*, *Bacillus*, and *Thermoascus* in Jiang-flavor types, supporting region-specific flavor formation mechanisms (5, 6). Despite these insights, most existing work has focused either on cross-regional comparisons or temperature effects, with fewer studies systematically evaluating the influence of storage or aging on microbial succession and function.

With the advancement of multi-omics technologies, the integrated analysis of microbial communities, functional genes, and metabolites has become an effective strategy for unraveling the complex biological processes that drive fermentation. Multi-omics integration, including metagenomics, metaproteomics, and metabolomics, provides a powerful framework to connect microbial composition with biochemical transformations (7–9). Such approaches have already elucidated mechanisms of phenotype formation in diverse biological systems, from animal adaptation to microbial ecology (10, 11). Recent studies in fermented foods and Baijiu research have leveraged multi-omics to deepen mechanistic insights. For example, a systematic review of microbial interactions and fermented food quality highlights how integrated metagenomics and metabolomics analyses simultaneously resolve microbial succession and flavor compound biosynthesis pathways across diverse fermented matrices, enabling targeted modulation of flavor and nutritional attributes (12). In the context of Baijiu, a study applying machine learning combined with metagenomics and flavoromics identified microbial and metabolic biomarkers underlying abnormal stacking fermentation in sauce-flavor Baijiu, revealing key taxa and metabolite signatures associated with fermentation dysregulation and aroma imbalance (13). Applying this framework to Daqu allows a more comprehensive understanding of how microbial structure and function jointly determine flavor precursor accumulation and fermentation quality.

Here, we conducted a systematic multi-omics investigation of new and aged Daqu collected from four representative Maotai-flavor liquor production regions in Guizhou Province (Maotai, Jinsha, Zunyi, and Xishui). Using shotgun metagenomic sequencing and untargeted LC-MS/MS metabolomics, we analyzed the microbial taxonomy, functional gene composition, and metabolite profiles of 48 Daqu samples. By integrating these data sets, we aimed to elucidate (i) how regional factors shape microbial communities and functional potential; (ii) how storage aging drives microbial succession and metabolite transformation; and (iii) how microbial–metabolite associations underlie region-specific flavor precursor formation. These results provide mechanistic insight into terroir-driven microbial ecology in Maotai-flavor liquor fermentation and offer a scientific basis for Daqu quality control and process optimization.

## RESULTS

### Genome-resolved and metabolomic profiling of Daqu samples

**Fig 1:**
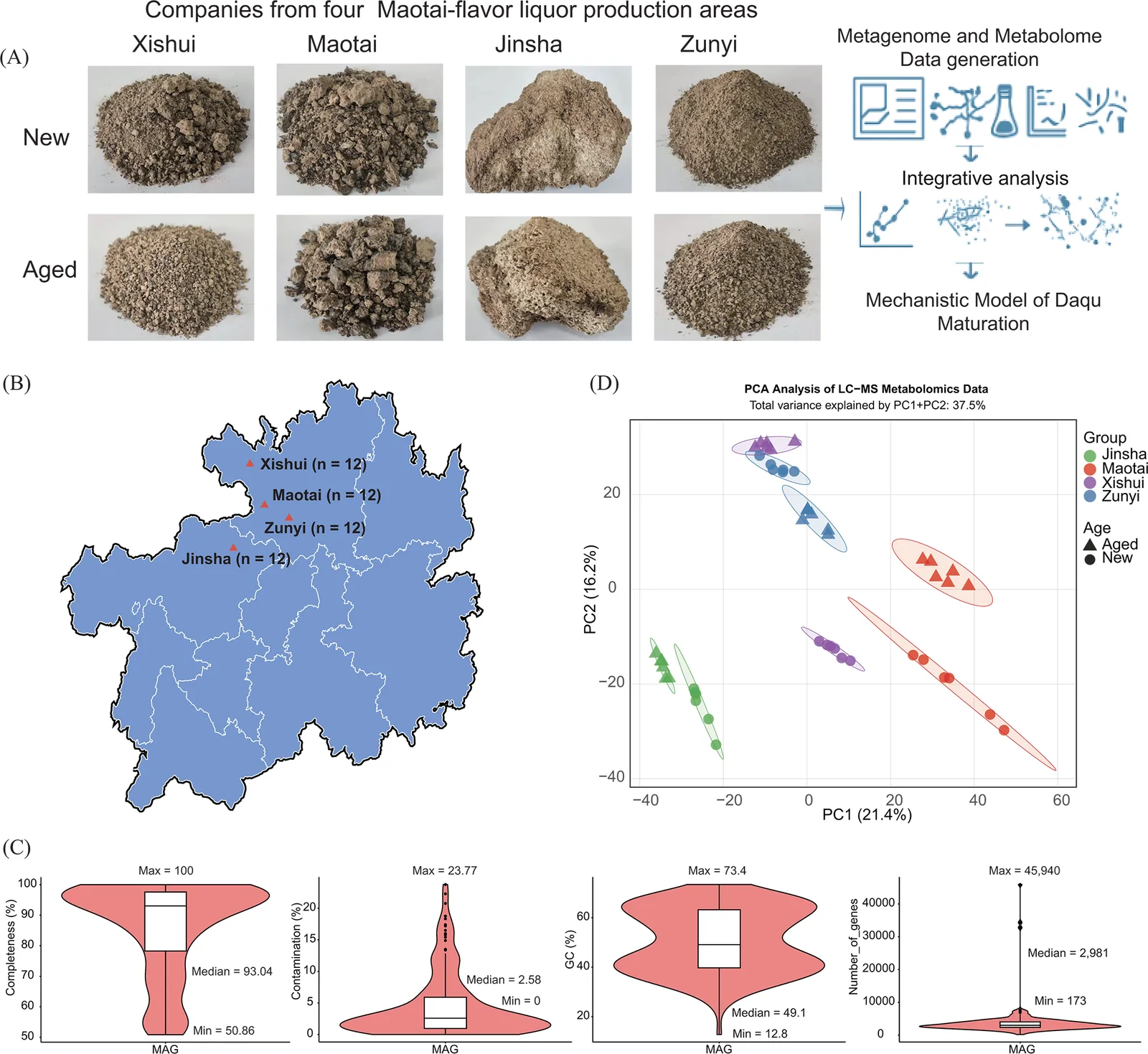
Regional sampling, MAGs quality assessment, and LC-MS Metabolomics Profile. (**A**) Representative photographs of new (freshly produced) and aged (6-month stored) Daqu collected from four major Maotai-flavor liquor-producing regions in Guizhou Province. (**B**) Map of sampling locations. The study included samples from four distinct regions: Jinsha, Maotai, Xishui, and Zunyi (*n* = 12 per location). (**C**) Quality assessment of MAGs. Violin and box plots display the distribution of key quality metrics for all recovered MAGs, including completeness (%), contamination (%), GC content (%), genome size (base pairs), contig N50 (base pairs), number of contigs, and number of genes. The median, maximum, and minimum values for each metric are provided. (**D**) PCA of LC-MS metabolomics data (*n* = 48).

To investigate how geographic origin and storage aging shape the microbial and metabolic architecture of Maotai-flavor liquor, a total of 48 samples were collected from four representative Maotai-flavor liquor-producing regions in Guizhou Province—Maotai (GT), Jinsha (JS), Zunyi (ZJ), and Xishui (AJ)—each comprising six biological replicates of both new and six-month-aged Daqu (Fig. 1A and B). Shotgun metagenomic sequencing yielded an average of 22.37 Gb of clean data per sample, with >97% of bases >Q30, confirming high technical quality (Table S1). After assembly, 163 (including 151 Bacteria and 12 Archaea genomes) non-redundant metagenome-assembled genomes (MAGs) were reconstructed with an average completeness of 86.10% and contamination <5% (MAG completeness ranged from 50.86% to 100%, with contamination ranging from 0% to 23.77%) (Fig. 1C). These genomes spanned 16 bacterial and 3 archaeal phyla, predominantly Bacillota, Pseudomonadota, and Actinomycetota (Table S2). Parallel untargeted LC-MS/MS metabolomics identified 2,642 molecular features across positive and negative ionization modes. Principal component analysis (PCA) indicated clear separation of new vs. aged Daqu in each location along PC1 and PC2, explaining ~37.6% of total variance (Fig. 1D). Collectively, the integrated genome-resolved and metabolomic data sets provided a comprehensive resource to decipher microbial succession and flavor-related metabolism in Daqu of Maotai-flavor liquor.

### Regional and aging-driven microbial community structures at the metagenome assembled genome level

**Fig 2:**
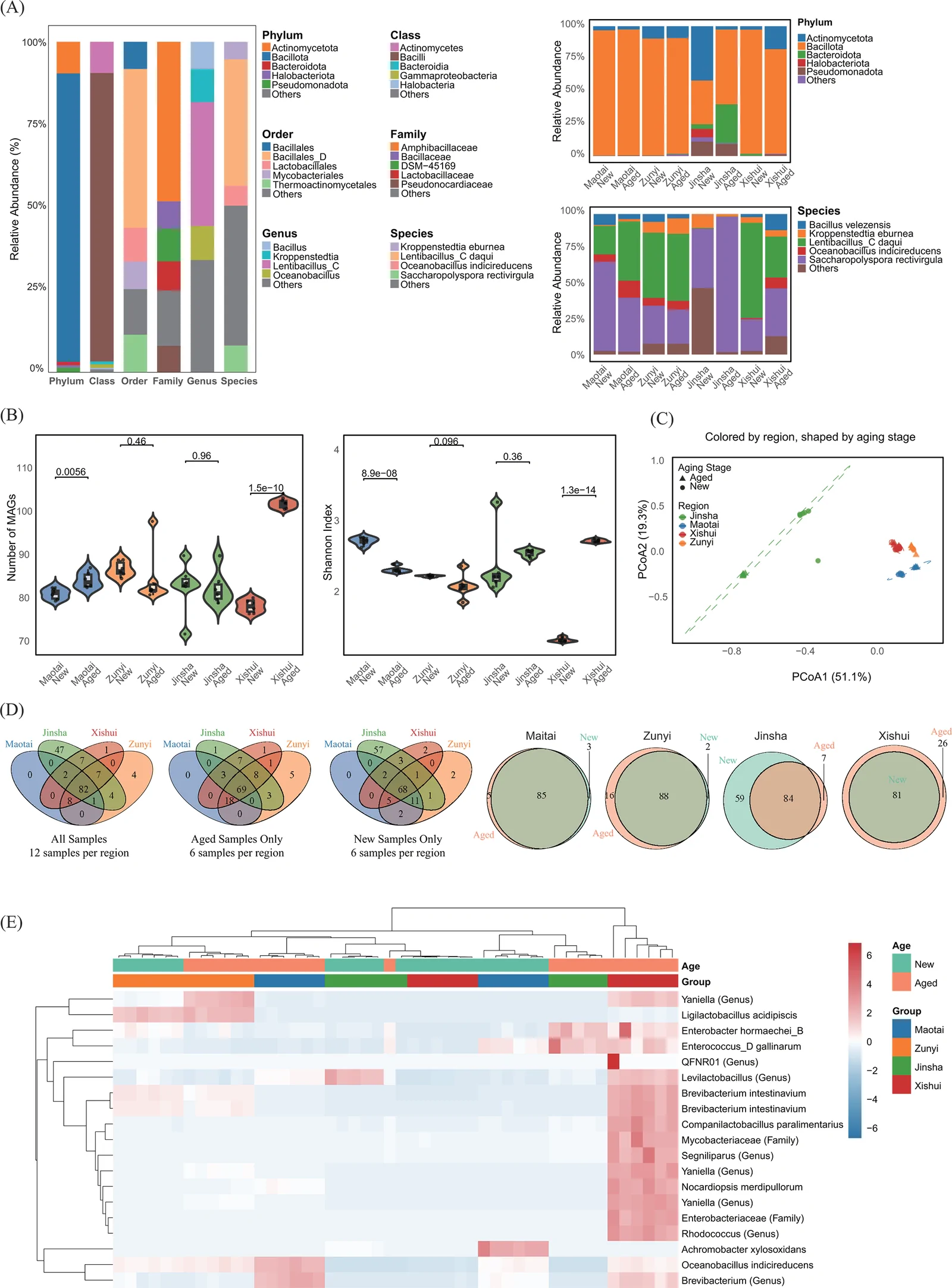
Microbial community structure, diversity, and differential abundance of MAGs. (**A**) Taxonomic composition of MAGs across regions and aging stages at multiple taxonomic levels. (**B**) Alpha diversity (observed richness and Shannon index) grouped by region and aging stage, with statistical significance indicated. (**C**) Beta diversity shows community differentiation among the four regions. (**D**) Venn diagrams showing shared and unique MAGs across regions and aging stages. (**E**) Heatmap of 19 MAGs with significantly different abundances between aged and new Daqu samples of Maotai-flavor liquor.

Having established the overall genomic and metabolic landscape of Daqu, we next explored how microbial community structures differ across regions and Daqu aging stages. Genome abundance profiles derived from the 163 MAGs revealed marked shifts in community composition during aging and across regions (Table S1). At the phylum level, Bacillota dominates at 87.21%, with Actinomycetota as the secondary phylum (9.56%), forming a highly specialized bacterial community. At the genus level, the taxa with the highest relative abundances were *Lentibacillus_C* (37.22%), *Oceanobacillus* (10.25%), *Kroppenstedtia* (9.87%), *Bacillus* (8.19%), and *Saccharopolyspora* (7.90%). At the species level, *Lentibacillus_C daqui* is the most abundant species (37.22%), followed by multiple co-dominant species, including *Saccharopolyspora rectivirgula* (7.90%) and *Oceanobacillus indicireducens* (5.82%) (Fig. 2A). The robustness of the microbial community structure is evidenced by its consistent profile, even as the relative abundances of constituent species vary with region and storage aging stage (Fig. 2B). Aging significantly increased both microbial richness and diversity (Shannon index) across regions, with the most pronounced effect observed in Jinsha (Fig. 2B). The PCoA plot revealed distinct microbial community structures between new and aged samples (Fig. 2C). Sequential PERMANOVA identified geographic region as the primary determinant of microbial composition, explaining 60.50% of total variance (*P* = 0.001). In contrast, the aging stage contributed only 3.92% of variance despite reaching statistical significance (*P* = 0.006). The analysis accounted for 64.42% of the total community variation, with regional effects demonstrating 15-fold greater influence than aging processes on microbial community structure (Table 1).

**TABLE 1:** Variance partitioning of microbial community composition by geographic region and aging stage using sequential PERMANOVA

Factor	Contribution percent	*R* squared	*P* value
Region	60.4956765	0.60495676	0.001
Aging stage	3.9243754	0.03924375	0.006
Residual	35.5799481	0.35579948	

A Venn diagram of MAG presence revealed 82 (50.31%) core MAGs shared across all regions and 52 (31.90%) unique MAGs restricted to specific locales (Fig. 2D). Zunyi and Jinsha harbor distinct region-specific MAGs, with Jinsha exhibiting exceptional microbial diversity (47 unique MAGs), while Maotai shows complete overlap with other regions and Xishui maintains only one (Enterobacterales_MAG2) unique MAG. Aged samples from Maotai, Zunyi, and Xishui all contained a higher number of unique MAGs compared to their new counterparts (Fig. 2D). A total of 19 MAGs showed significant abundance differences between new and aged groups, with only one decreasing in aged samples and the remaining 18 exhibiting increased abundance (Fig. 2E). These findings confirm that both terroir and storage aging jointly modulate genome-level microbial assemblages in Maotai-flavor liquor.

### Functional potential, resistance, and mobile genetic elements of Daqu microbiomes

**Fig 3:**
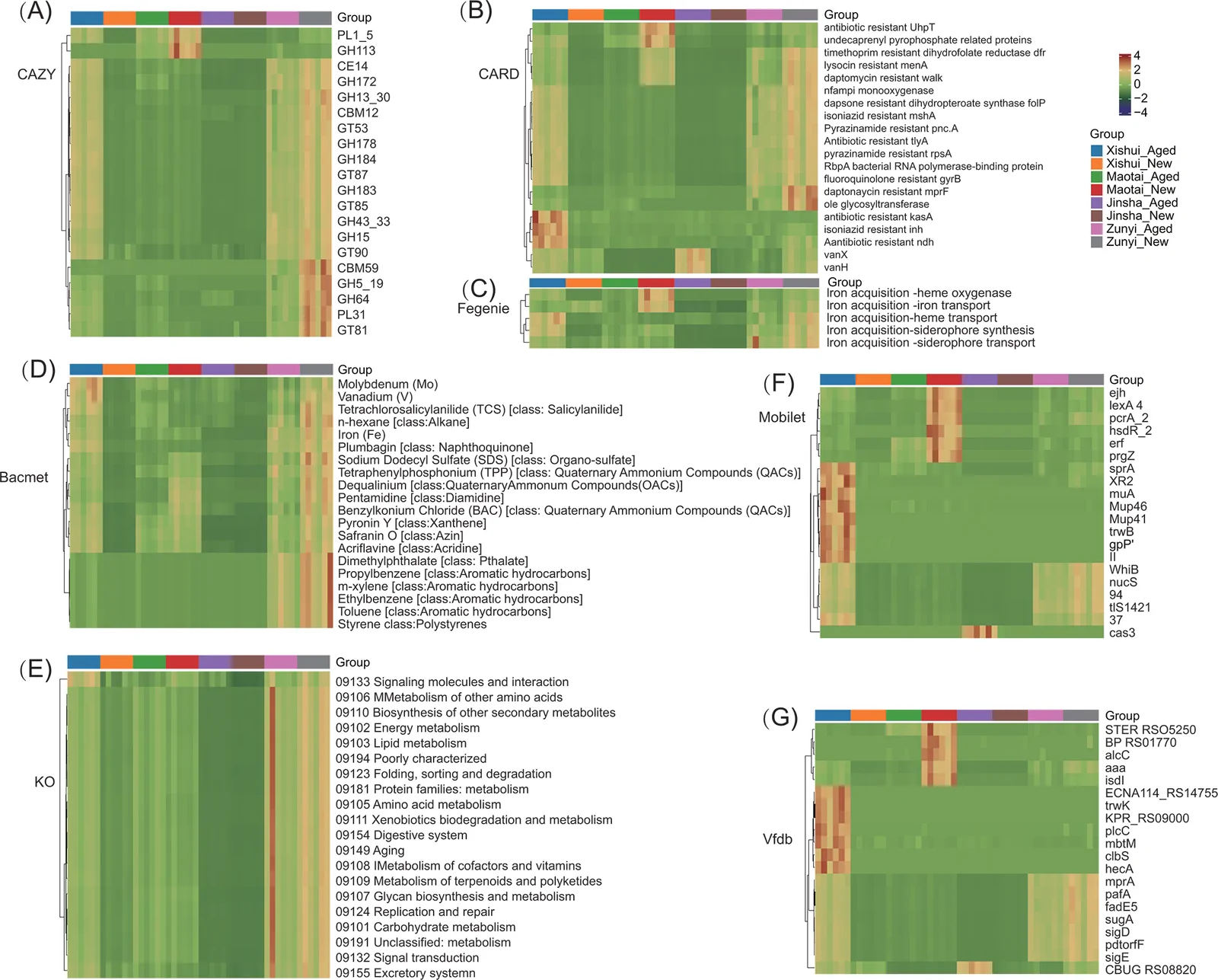
Functional repertoire and adaptive traits of Daqu microbiomes. Heatmaps for CAZy family (**A**), antibiotic resistance genes (**B**), heavy metal and xenobiotic resistance genes (**C**), iron acquisition systems (**D**), KEGG enrichment (**E**), mobile genetic elements (**F**), and virulence-related genes (**G**) distribution in new and aged Daqu samples.

Beyond community composition, understanding the functional capacity of the microbiome is critical for interpreting its biochemical roles during fermentation. Comprehensive functional annotation of the metagenomic data against multiple databases (KEGG, CAZy, VFDB, Fegenie, CARD, mobileOG-db, and so on) revealed that both new and aged Daqu samples possessed metabolically versatile and ecologically complex microbial communities. The microbiome harbors an extensive arsenal of carbohydrate-active enzymes (CAZy), predominantly glycoside hydrolases (GH13, GH43, and GH3), glycosyl transferases, carbohydrate esterases, and polysaccharide lyases, which collectively drive the efficient degradation of complex plant polysaccharides (starch, cellulose, and hemicellulose) into fermentable sugars (Fig. 3A). This robust substrate-deconstructing capability forms the fundamental basis for subsequent flavor precursor generation. Beyond core metabolism, the Daqu ecosystem possesses a sophisticated defense and adaptation machinery. A diverse resistome was identified, encompassing genes conferring resistance to multiple antibiotic classes, including rifampin, daptomycin, and trimethoprim (Fig. 3B), likely reflecting intrinsic environmental resistance and long-term microbial competition within the solid-state fermentation matrix. Iron acquisition systems were highly developed, with complete genetic modules for siderophore synthesis and transport, heme uptake, and heme oxygenase activity, ensuring sufficient iron availability—a crucial micronutrient for microbial growth and enzymatic activities (Fig. 3C).

Furthermore, genes involved in detoxification and resistance to heavy metals and xenobiotics, such as molybdenum, pyronin Y, and safranin O, were prevalent, highlighting the community’s resilience to chemical stressors (Fig. 3D). The functional potential, as per KEGG annotation, was heavily enriched in core metabolic pathways like carbohydrate metabolism, amino acid metabolism, energy metabolism, and lipid metabolism, alongside significant representation of biosynthesis of other secondary metabolites, directly linking the microbiome to the production of diverse flavor and aroma compounds (Fig. 3E). Notably, the presence of various mobile genetic elements, such as transposases (e.g., *tIS1421*, *muA*, *Mup41*/*Mup46,* and *XR2*) and integrases (*erf* and *trwB*), suggests active horizontal gene transfer potential, facilitating microbial adaptation and functional diversification (Fig. 3F). Interestingly, virulence factor genes related to adhesion, secretion systems, and biofilm formation were detected; in the fermentation context, these likely aid in microbial colonization, community stability, and niche competition rather than pathogenicity (Fig. 3G). In summary, the Daqu microbiome of Maotai-flavor liquor is characterized by its potent substrate-saccharifying capacity, multifaceted stress resistance, efficient nutrient scavenging, and dynamic genetic plasticity. These interconnected functional traits ensure efficient raw material transformation and uphold ecosystem stability during the aging process, collectively establishing the essential biochemical foundation for the complex and distinctive flavor profile of Maotai-flavor liquor. The aging process appears to further refine these functionalities, particularly by enhancing CAZy diversity and mobilome activity, which correlate with the superior fermentation performance traditionally attributed to aged Daqu.

### Cellulolytic MAGs and their contribution to polysaccharide degradation

Comprehensive functional annotation of the MAGs was also conducted. Analysis of the reconstructed MAGs revealed that several taxa with well-established cellulolytic or hemicellulolytic capabilities were enriched in Daqu, especially in aged samples (Table S4 and S5). Actinomycetota MAGs, including *Nocardiopsis meridipullorum*, *Saccharopolyspora rectivirgula*, *Streptomyces cacaoi*, and *Kroppenstedtia spp*., are known producers of extracellular cellulases and xylanases and encode multiple GH43, GH3, and other CAZyme families associated with lignocellulose depolymerization (Fig. S1). Among *Bacillota*, MAGs of *Bacillus licheniformis*, Bacillus velezensis, *Bacillus sonorensis*, *Weizmannia coagulans*, and *Thermoactinomyces spp*. carried GH13, GH43, and GH3 enzymes and likely drove amylolytic and hemicellulolytic activities under high-temperature Daqu conditions (Table S6). The dominant *Lentibacillus_C daqui* MAG also contained GH43 and GH3 members, consistent with its putative saccharifying role in high-temperature and saline environments. Additional genera such as *Paenibacillus*, *Planifilum*, *Burkholderia*, *Pantoea*, and *Kosakonia* may serve as auxiliary cellulose/hemicellulose degraders. Together, these cellulolytic MAGs form a functional consortium that enables efficient conversion of plant-derived polysaccharides into fermentable sugars, supporting subsequent aroma precursor formation during Daqu maturation.

### Untargeted metabolomics reveals distinct regional and aging signatures

**Fig 4:**
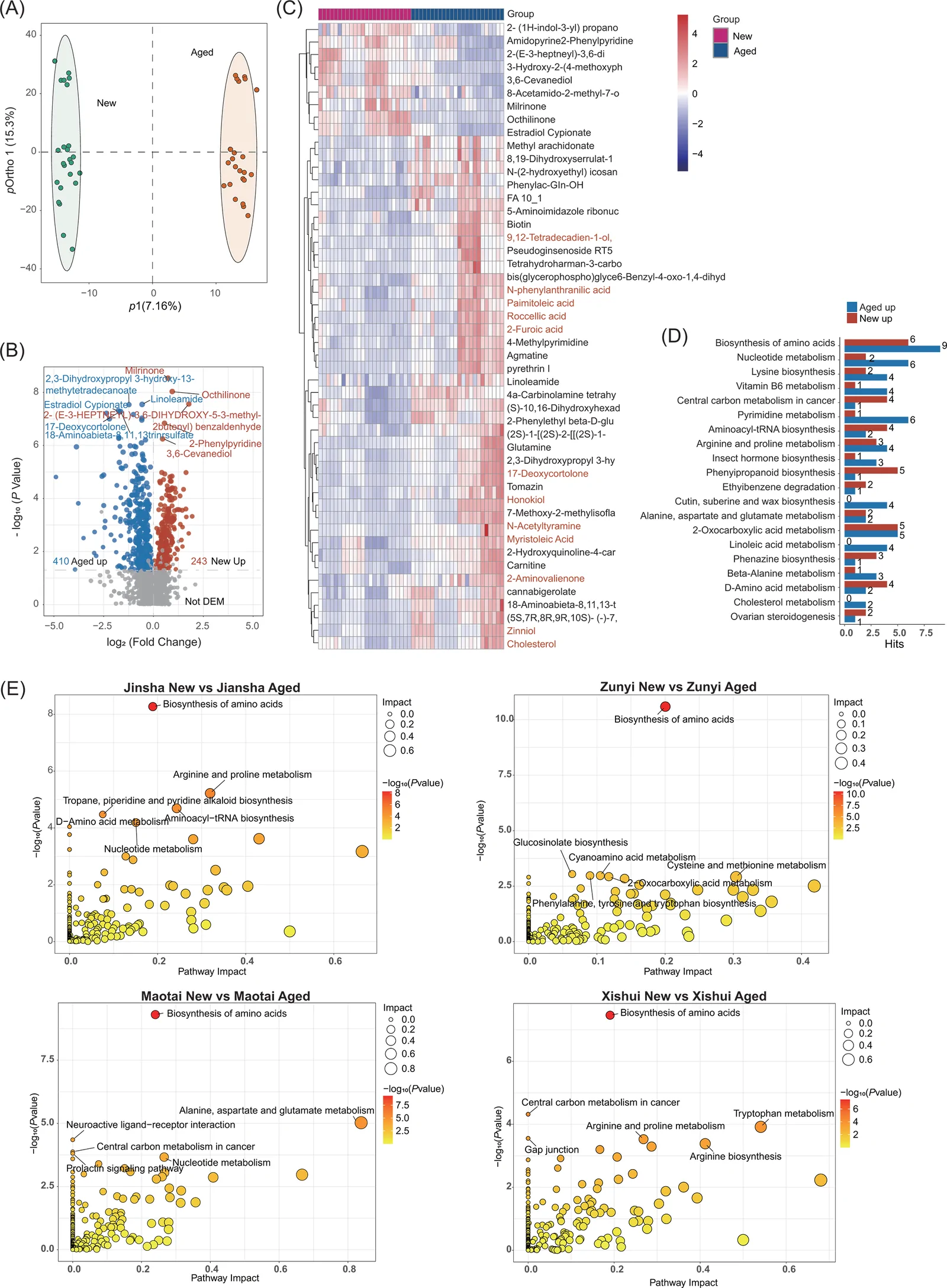
Differential metabolomic profiles between new and aged Daqu. (**A**) OPLS-DA score plot showing clear separation between new and aged Daqu based on metabolite profiles. (**B**) Volcano plot of differential metabolites (DEMs); red points represent metabolites upregulated in aged Daqu (log₂FC > 1, *P* < 0.05). (**C**) Heatmap of the top 50 significantly different metabolites, illustrating consistent upregulation of esters, fatty acids, and aromatic compounds in aged samples. Important metabolites are highlighted. (**D**) KEGG pathway enrichment of DEMs, highlighting predominant enrichment in amino acid, fatty acid, and secondary metabolite metabolism. (**E**) KEGG pathway enrichment analysis comparing new and aged Daqu from four production regions. The x-axis represents pathway impact, and the y-axis indicates significance (−log₁₀ *P* value). Bubble size corresponds to relative pathway impact.

Given that microbial activity ultimately manifests as metabolite production, we next characterized the metabolomic profiles of new and aged Daqu used in Maotai-flavor liquor. Untargeted LC-MS/MS metabolomics identified 2,642 molecular features across both positive and negative ionization modes (Table S7). Principal component analysis demonstrated clear separation of Daqu samples by region and aging stage (Fig. 1C). OPLS-DA analysis showed a clear separation between the two groups (*R*²*Y* = 0.994, *Q*² = 0.909), indicating robust discrimination of aging stages (Fig. 4A). A total of 653 differential metabolites were identified, among which 243 were upregulated in new Daqu (Fig. 4B), 410 were upregulated in aged Daqu. The heatmap of the top 50 differential features (Fig. 4C, Fig. S2) highlighted enrichment of flavor-related compounds such as ethyl esters and aromatic alcohols in aged samples. The differential metabolites enriched in aged Daqu mainly include fatty acids, phenolic compounds, amino acid derivatives, and steroid-like molecules. These metabolites participate in lipid oxidation, amino acid degradation, and secondary metabolism, contributing to enhanced ester, pyrazine, and phenolic aroma formation. Representative compounds included typical ethyl esters (e.g., ethyl acetate, ethyl lactate, and ethyl caproate), amino acid–derived metabolites (such as phenethyl alcohol and other aromatic compounds), and organic acids including lactic acid and acetic acid. These molecules are important contributors to ester aroma, floral notes, and acidity balance in Maotai-flavor Baijiu. Collectively, they reflect intensified microbial activity and metabolic diversification that underpin the complex flavor maturation of aged Daqu. KEGG enrichment analysis (Fig. 4D) indicated that upregulated metabolites in aged Daqu were predominantly associated with biosynthesis of amino acids, linoleic acid metabolism, and nucleotide metabolism, all contributing to the formation of key aroma precursors.

Comparative KEGG enrichment analysis revealed both shared and region-specific metabolic transitions between new and aged Daqu across the four production regions (Fig. 4E). Biosynthesis of amino acids was consistently enriched in all comparisons, indicating that amino acid metabolism is a central biochemical process during Daqu maturation. In Jinsha Daqu, significant enrichment was observed in arginine and proline metabolism, aminoacyl-tRNA biosynthesis, and nucleotide metabolism, reflecting intensified protein synthesis and nitrogen turnover during aging. Zunyi Daqu exhibited enrichment in aromatic amino acid biosynthesis and sulfur amino acid metabolism, suggesting enhanced formation of aromatic and sulfur-derived flavor precursors. In Maotai Daqu, pathways related to tryptophan metabolism, arginine metabolism, and central carbon metabolism were predominant, indicating coupled regulation of amino acid catabolism and energy metabolism. Xishui Daqu showed similar enrichment in arginine biosynthesis and central carbon metabolism. Collectively, these results demonstrate that aging promotes amino acid biosynthetic and energy-related pathways, highlighting intensified nitrogen and carbon flux during Daqu maturation.

### The integrative analysis of bacterial community profiles and metabolomic data across different Daqu samples

**Fig 5:**
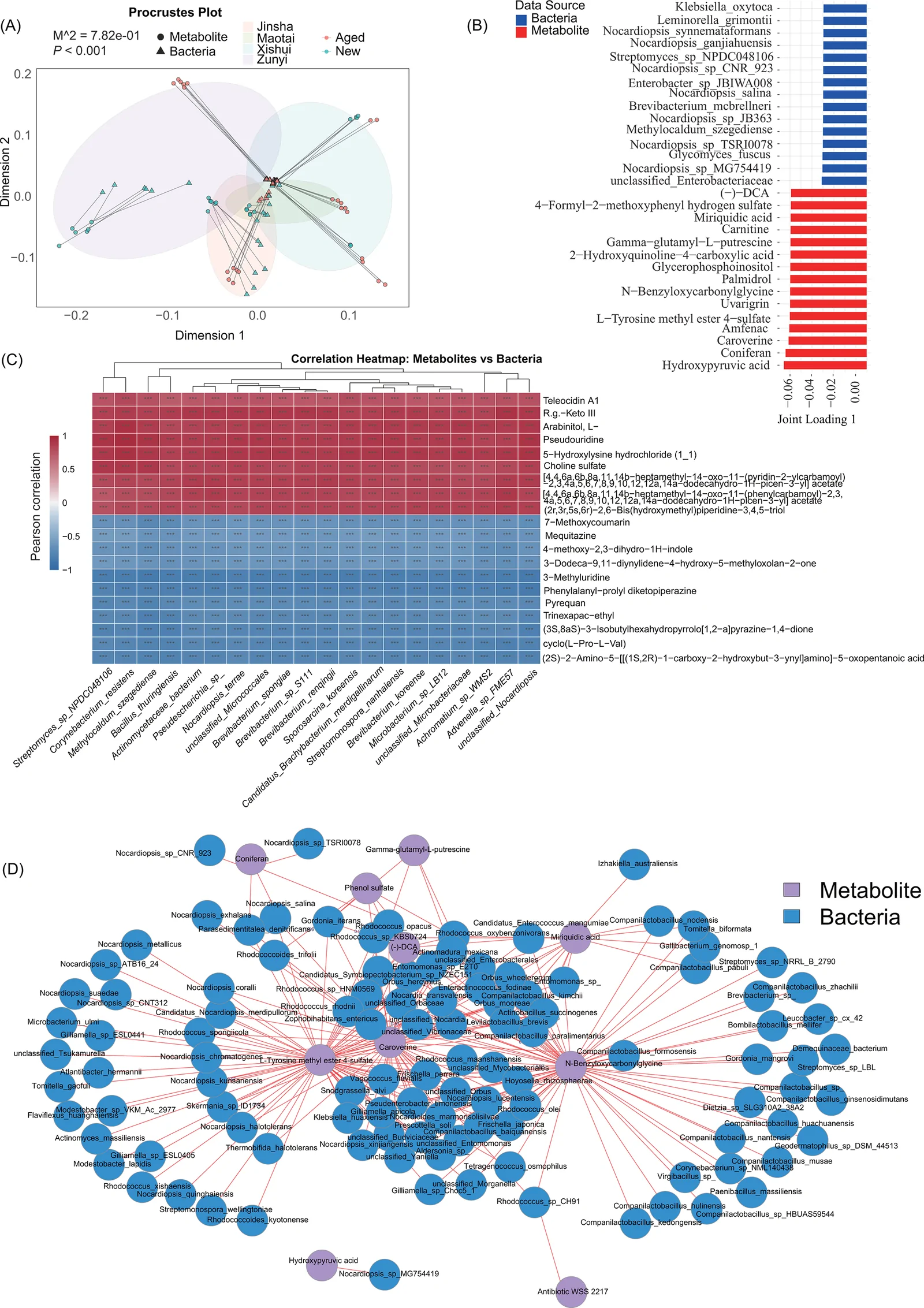
Integration of metabolomic and microbiomic data across Daqu fermentation samples from four Maotai-flavor liquor regions. (**A**) Procrustes analysis showing the concordance between metabolite and bacterial community structures. *M*² = 0.782, *P* < 0.001. (**B**) Joint loadings from PLS integration analysis showing contributions of metabolites and bacteria to the first dimension. (**C**) Correlation heatmap displaying associations between specific metabolites and bacterial taxa. Spearman correlation was used for bacteria–metabolite associations. *P*-values were adjusted using Benjamini–Hochberg correction. Significant correlations were defined as *P* < 0.05. (**D**) Detailed view of key metabolite-bacteria correlations, highlighting specific compounds and microbial species with strong associations.

Procrustes analysis shows a significant concordance between microbial composition and metabolite profiles (*M*² = 0.782, *P* < 0.001) (Fig. 5A). This strong alignment indicates that variations in bacterial communities are closely associated with shifts in metabolite patterns. Samples cluster according to production regions (Jinsha, Maotai, Xishui, and Zunyi) and Daqu age, suggesting that both geographical origin and fermentation maturity influence the joint microbiome–metabolome structure. The correlation heatmap reveals extensive and significant correlations between specific bacterial taxa and individual metabolites. Notably, genera such as *Nocardiopsis*, *Streptomyces*, *Rhodococcus*, and *Companilactobacillus*, species like *Bacillus thuringiensis*, *Actinomycetaceae bacterium,* and *Methylocaldum szegediense* are significantly associated with key small molecules, including amino-acid derivatives, nucleosides, aromatic compounds, and bioactive secondary metabolites (Fig. 5B and C). These patterns indicate that dominant functional taxa play crucial roles in shaping the chemical profile of Daqu. Joint loadings from the integrative multivariate model identify groups of bacteria and metabolites that are significantly associated with. Several *Nocardiopsis* species, *Streptomyces* strains, and *Rhodococcus* bacteria align with metabolites such as hydroxypyruvic acid, gamma-glutamyl-L-putrescine, and Antibiotic WSS 2217, suggesting coordinated metabolic interactions during fermentation. These paired loadings further reinforce that specific microbes are likely responsible for the biosynthesis, transformation, or degradation of characteristic Daqu metabolites. L−Tyrosine methyl ester 4-sulfate, N-Benzyloxycarbonylglycine, Caroverine, (−) −DCA, and Miriquidic acid align with an abundant bacteria (Fig. 5D).

**Fig 6:**
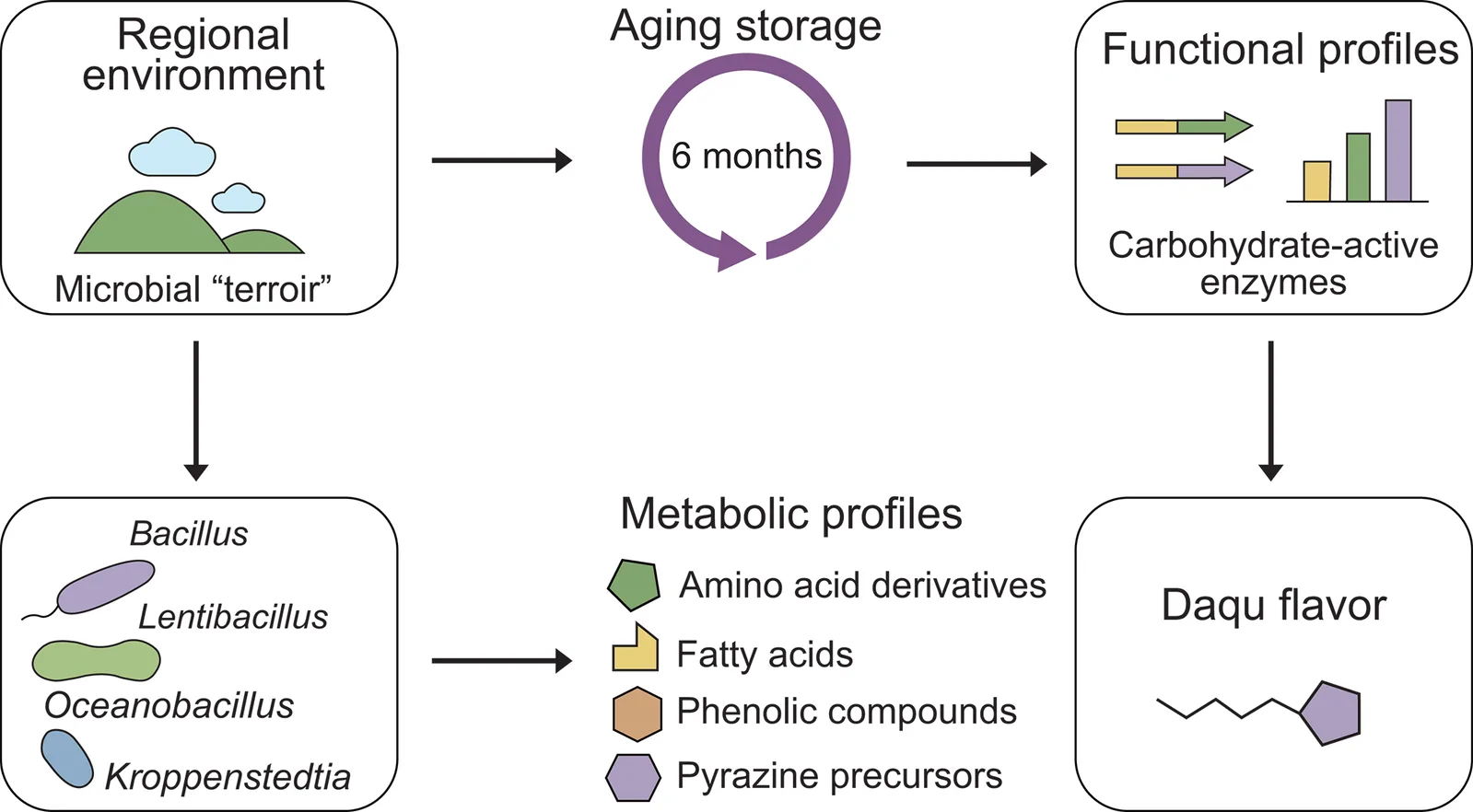
Schematic model of multi-omics integration illustrating microbial–metabolite–flavor coupling during Daqu maturation of Maotai-flavor liquor.

In total, regional environmental factors (microbial “terroir”) shape the initial community structure, introducing distinct sets of Bacillota- and Actinomycetota-dominated taxa (Fig. 6). During 6 months of storage, aging drives pronounced microbial succession, characterized by increased abundance of thermophilic and spore-forming genera such as *Bacillus*, *Kroppenstedtia*, *Lentibacillus_C*, and *Oceanobacillus*. These taxa contribute to enhanced functional capacities, including elevated carbohydrate-active enzymes (GH13, GH43, and GH3), intensified amino acid and lipid metabolism, and expanded secondary metabolic pathways. The integration of metagenomic and metabolomic data sets demonstrates that these functional shifts directly translate into metabolic remodeling. Key metabolites—amino acid derivatives, fatty acids, phenolic compounds, and pyrazine precursors—show strong correlations with dominant MAGs, forming coordinated microbe–metabolite modules. Together, the model highlights how terroir-driven microbial assembly, aging-induced functional specialization, and metabolite accumulation converge to shape the production of characteristic ester, pyrazine, and phenolic aroma precursors that underpin the flavor quality of Maotai-flavor liquor.

## DISCUSSION

This study provides an integrated multi-omics view of the microbial and metabolic dynamics underlying the maturation of Daqu from four representative Maotai-flavor liquor production regions in Guizhou Province. Through genome-resolved metagenomics and untargeted metabolomics, we revealed that both regional origin and storage aging jointly shape the microbial community composition, functional gene repertoire, and metabolite profiles of Daqu, ultimately defining its fermentation potential and flavor foundation.

### Taxonomic classification and ecological insight of MAGs

In the present study, MAGs recovered from Daqu exhibited diverse phylogenetic assignments, predominantly within the phyla Bacillota and Actinomycetota (data/GTDB assignments). This taxonomic profile is consistent with previous metagenomic investigations of high-temperature Daqu (14), in which Firmicutes/Bacillota lineages dominate at both the phylum and genus levels and contribute key functions in saccharification and fermentation processes (e.g., *Bacillus*, *Saccharopolyspora*, *Pediococcus*) as revealed by short-read and genome-centric analyses. Recently, Lv et al. (15) reconstructed MAGs from high-temperature Daqu and demonstrated that three MAGs form a monophyletic clade within the genus *Pseudogracilibacillus*, distinct from known type strains based on average nucleotide identity and phylogenomics, leading to the proposal of a novel species (*Pseudogracilibacillus amylolyticus sp. nov.*) (15). This finding expands the known taxonomic diversity of Daqu microbiomes and exemplifies the power of MAGs to identify previously uncultured lineages with potential ecological roles in starch and aroma compound degradation. In the present study, 71 MAGs could not be assigned to a known species, likely due to the limited availability of closely related reference genomes in public databases, suggesting that Daqu harbors substantial unexplored microbial diversity and potentially novel taxa that remain to be taxonomically characterized. Comparative studies in other fermentation systems further support the utility of MAG reconstruction for defining predominant taxa that may be overlooked by 16S rRNA amplicon profiling alone. For instance, in vinegar fermentation microbiomes, the recovery of hundreds of MAGs revealed *Acetilactobacillus*, *Lactobacillus*, *Weizmannia,* and others as leading species-level genomes linked to functional potential in acid production (16). Similarly, MAG-based surveys in cocoa fermentation uncovered lactic acid bacteria, acetic acid bacteria, and *Bacilli* genomes that clarified community structure and metabolic capabilities beyond OTU-level taxonomies (17). Together, these studies indicate that integrating MAG taxonomy with ecological context is critical for understanding microbial community structure in complex fermentations like Daqu.

### Regional differentiation of Daqu microbiomes

The pronounced regional divergence in microbial assemblages observed here supports the concept of Baijiu “microbial terroir,” in which local environments select for distinct microbial consortia and enzymatic functions (3, 5). Although Bacillota and Actinomycetota dominated all samples, Jinsha Daqu exhibited the highest genomic diversity and number of unique MAGs, indicating stronger environmental selection and microbial recruitment from its local production ecosystem. In contrast, Maotai Daqu shared nearly complete overlap with other regions, reflecting homogenization due to long-established production practices. Such region-dependent microbial differentiation parallels recent reports showing that Daqu from different provinces harbor unique thermophilic bacterial and fungal taxa linked to characteristic flavor styles (2, 4).

### Microbial succession and functional adaptation during aging

Aging significantly increased microbial richness and functional gene diversity, indicating ecological stabilization and metabolic specialization during storage. The enrichment of thermophilic and spore-forming taxa, such as *Bacillus*, *Lentibacillus*, and *Thermoactinomyces,* enhances enzymatic robustness under the high-temperature fermentation conditions typical of sauce-aroma Baijiu (2, 18). Functional annotation revealed that aged Daqu exhibited a higher abundance of genes involved in carbohydrate, amino acid, and energy metabolism, consistent with its superior fermentation performance recognized by industry practices. The elevated diversity of glycoside hydrolase (GH) families—especially GH13, GH43, and GH3—suggests intensified polysaccharide degradation and sugar release during maturation, aligning with observations in other solid-state fermentations where carbohydrate metabolism is a key driver of microbial succession (9, 19). A striking feature of GH43 enzymes is their bi-functionality, with several members exhibiting both β-xylosidase and α-arabinofuranosidase activities (20).

Members of the genus *Bacillus* (including *B. licheniformis* and *B. subtilis* relatives) are repeatedly implicated in pyrazine biosynthesis and in promoting ester formation in solid-state fermentations (21–23). Experimental inoculation studies show that fortifying Daqu with *B. licheniformis* markedly increases total volatile compounds and strongly elevates pyrazine levels (such as precursor 2,3-butanediol). Consistent with this previous study (24), our observation was that *Bacillus* MAGs were also co-associated with pyrazine precursors. The mechanistic basis includes stimulation of nitrogen metabolism (providing ammonium and amino-carbon fragments) and production of 2,3-butanediol and related intermediates that condense into alkylpyrazines under thermal conditions typical of Daqu processing (24). Additionally, *Bacillus spp.* produce extracellular hydrolases and esterifying activities that can increase ester formation either directly (microbial ester synthases/esterases) or indirectly by releasing alcohols and acyl-CoA pools that chemical or enzymatic esterification converts to ethyl esters—metabolites we found enriched in aged Daqu (25). These roles are supported by studies describing Bacillus-driven pyrazine biosynthesis in diverse fermented foods and the capacity of Bacillus strains to modulate ester profiles (24–26).

### Metabolic remodeling and flavor precursor formation

Metabolomic profiling confirmed that aging promotes the accumulation of amino acid derivatives, fatty acids, esters, and phenolic compounds, which are essential flavor precursors in Maotai-flavor liquor. KEGG enrichment of aged Daqu in amino acid degradation, linoleic acid metabolism, and phenylpropanoid biosynthesis pathways implies that amino acid catabolism and lipid oxidation jointly contribute to enhanced pyrazine, ester, and phenolic aroma formation. The integration of metagenomic and metabolomic data revealed that *Bacillus licheniformis* encodes complete pyrazine synthesis modules, supporting their central role in generating characteristic sauce-like volatiles (27). These findings support a shift in metabolic focus from substrate hydrolysis in new Daqu toward secondary metabolism and aroma compound biosynthesis in aged Daqu.

*Lentibacillus* (including taxa recently isolated from Daqu) is halotolerant/thermotolerant Firmicutes often abundant in high-temperature starters (28). Their physiology is consistent with survival and activity in the nutrient-rich, saline microenvironments of Daqu, and they are plausible contributors to carbohydrate hydrolysis and amino acid turnover under those conditions. The isolation of *Lentibacillus daqui* from Daqu confirms this genus as a resident taxon in such systems (29); their metabolic repertoire likely complements Bacillus and Thermoactinomyces activities during aging and stabilizes community function under stress. *Kroppenstedtia eburnea*, *Lentibacillus daqui*, and *Bacillus* species were the main contributors to these enzymes related to fermenting, esterifying, and liquefying powers, crucial for high-temperature Daqu quality (29).

In addition, *Nocardiopsis* and related actinomycetes are prolific producers of secondary metabolites (including polyketides, alkaloids, and phenolic derivatives) and possess diverse biosynthetic gene clusters (30). Their enrichment and strong correlations with certain phenolic and antibiotic-like metabolites in our data suggest they contribute to the chemical diversity of aged Daqu, possibly by synthesizing bioactive small molecules or transforming plant-derived phenolics (31). Beyond flavor chemistry, Nocardiopsis-derived metabolites may influence microbial interactions (antagonism or signaling) and therefore indirectly shape metabolite trajectories during maturation (30, 32). Furthermore, one clear functional outcome of aging in our data set is enrichment of GH families (notably GH13 and related α-amylase subfamilies), these enzymatic activities plausibly increase the substrate pool for microbial amino acid catabolism and volatile precursor production during aging (33, 34).

### Genetic plasticity and ecological resilience

The discovery of abundant transposases (e.g., *tIS1421*, *muA*, *Mup41*/*Mup46,* and *XR2*) and integrases (*erf*, *trwB*) indicates an active mobilome within Daqu microbiomes. This genomic flexibility likely accelerates horizontal gene transfer (HGT), facilitating rapid microbial adaptation and diversification of metabolic functions during storage. Such genetic mobility has been proposed as a major force sustaining functional stability in complex fermentation ecosystems (35). Concurrently, the detection of antibiotic and heavy-metal resistance genes, siderophore-mediated iron acquisition systems, and detoxification modules underscores the microbiome’s strong adaptive capacity to environmental stressors. Notably, virulence-related genes associated with adhesion and biofilm formation may enhance microbial colonization and interspecies cooperation rather than pathogenicity, contributing to community resilience under solid-state conditions (36, 37).

The detection of antimicrobial resistance (AMR) and virulence-associated genes in Daqu metagenomes does not imply active pathogenicity or food safety risk. These genes likely represent ecological adaptation mechanisms of environmental microorganisms under fermentation stress (38). Importantly, high-temperature fermentation and subsequent distillation in Baijiu production effectively eliminate viable cells, preventing resistance transmission and ensuring minimal food safety risk (39). Nevertheless, continued surveillance of AMR genes in fermentation environments within a One Health framework remains warranted (40).

### Formation of pyrazines, esters, and phenolics—integrated pathways

Pyrazines (e.g., tetramethylpyrazine and other alkylpyrazines) are considered signature volatiles in many Baijiu styles (41). Their biosynthesis in fermentation systems typically requires amino-nitrogen (ammonium), α-dicarbonyls or alcohol precursors (such as 2,3-butanediol), and heat-promoted condensation reactions (42); microbiota that elevate nitrogen turnover and produce 2,3-butanediol therefore favor pyrazine accumulation (24–26). Esters arise through enzymatic esterification (microbial alcohol acyltransferases/esterases) or chemical esterification of alcohols and acids; lactic acid bacteria and Bacillus have both been reported to contribute to ester synthesis in fermented foods, aligning with our co-occurrence of Companilactobacillus/other LAB and increased ester signals in aged Daqu (43). Finally, phenolic compounds—either liberated from bound plant-derived phenolics by microbial glycosidases/tannases or transformed by microbial oxidative enzymes—accumulate with aging and can contribute to mouthfeel and aroma complexity (44).

### Implications, limitations, and future directions

Collectively, our data support a model in which regional contribution and aging synergize: terroir defines initial community composition; aging enriches functional traits (CAZymes, nitrogen metabolism, esterases, secondary metabolism), which, in turn, remodel the metabolome to accumulate flavor precursors (pyrazines, esters, phenolics). This mechanistic insight suggests actionable strategies for starter management—for example, targeted augmentation with pyrazine-producing Bacillus strains or modulation of aging conditions to favor beneficial GH/esterase activities. However, causality remains to be validated experimentally: future work should couple isolation and characterization of key strains (functional assays for pyrazine/ester production, GH activity), stable isotope tracing to confirm microbial biosynthetic routes, and controlled inoculation/co-culture experiments to verify microbe contribution to metabolite causal chains. Understanding the genetics of biosynthetic loci (e.g., 2,3-butanediol pathway, pyrazine-forming modules, esterases) in the MAGs we recovered would also prioritize candidate genes for functional validation.

## MATERIALS AND METHODS

### Sample collection

Samples were collected from four major Maotai-flavor liquor production regions in Guizhou Province, China: Maotai, Jinsha, Zunyi, and Xishui. From each region, one representative distillery was selected for sampling. Both fresh *Daqu* (newly produced fermentation starter) and aged *Daqu* (stored for six months) were obtained. A total of 48 samples were collected, with 12 samples per region (6 fresh and 6 aged *Daqu*). Each sample weighed 50–60 g and was stored in sterile zip-lock bags. Samples were selected in June 2025 based on the following criteria: (i) consistent production process and raw material formulation, (ii) similar fermentation duration and maturity stage, and (iii) absence of visible contamination or structural damage. The selection aimed to capture regional characteristics while minimizing within-batch heterogeneity. All samples were immediately placed on ice after collection and transported under cold chain conditions to the laboratory for further analysis.

### DNA extraction and quality control

Genomic DNA was extracted from each sample using a commercial DNA extraction kit (Qiagen, Gaithersburg, MD, USA) suitable for environmental microbiomes. The quality and integrity of the extracted DNA were assessed via agarose gel electrophoresis to ensure clear bands without significant degradation. DNA concentration and purity were quantified using a Qubit 3.0 Spectrophotometer (Thermo Fisher Scientific, USA). Only DNA samples meeting the criteria of concentration ≥25 ng/µL and total mass ≥2 µg were used for subsequent library preparation.

### Library preparation

The AccuMetaG absolute quantification metagenomic sequencing approach was employed. A defined quantity of internal standard sequences was spiked into each qualified DNA sample. The mixture was subjected to random fragmentation using a Covaris ME220 system (Covaris, USA) to generate DNA fragments with a target size of 400 bp (range: 200–600 bp). The fragmented DNA underwent end repair, 3′-adenylation, and adapter ligation using the GS-DNA Library Preparation Kit (Shanghai GeneCowin, China). The ligated products were amplified via PCR with high-fidelity polymerase under limited cycles (≤8) to minimize amplification bias. The final libraries were quantified using Qubit, and their size distribution was verified to ensure fragments were 300–500 bp.

### Sequencing

The qualified libraries were sequenced on the DNBSEQ-T7 platform (MGI, China) using a 2 × 150 bp paired-end sequencing strategy. Raw sequencing data in FASTQ format were generated for downstream bioinformatic analysis.

### Bioinformatic analysis

Raw sequencing data were subjected to quality control using FastQC (v0.11.9) to assess base quality and composition. Low-quality reads, adapter sequences, and reads with ambiguous bases (*N* > 1) or length <100 bp were filtered out using fastp (v0.23.2) (45). High-quality clean reads were retained for subsequent analyses. Metagenomic assembly was performed using metaSPAdes (v4.2.0) (46), and non-redundant contigs and genes were constructed using MMseqs2 (v15.6f452) (47) and Prodigal (v2.6.3) (48), respectively. Taxonomic profiling and functional annotation were conducted using MetaPhlAn (v4.1.1) (49) and DIAMOND (v2.0.14) (50) against databases including COG, KEGG, and CAZy. Absolute quantification of genes was achieved by calibrating against the spike-in internal standards. Differential abundance analysis of species and functional pathways was performed using statistical methods implemented in R (v4.3). Data visualization was carried out using ggplot2 and Circos (v0.69-6).

### Microbial community analysis

Microbial community analysis was performed on 163 MAGs across four regions (Maotai, Zunyi, Jinsha, Xishui) with new and aged samples. Taxonomic composition was visualized using stacked bar plots at six taxonomic levels, displaying the top five taxa with others grouped. Alpha diversity (Shannon index and observed richness) was calculated using the vegan package and visualized with violin plots containing embedded boxplots, with statistical significance assessed by t-tests. Beta diversity analysis employed Bray-Curtis distances with PCoA visualization and PERMANOVA (Permutational Multivariate Analysis of Variance) testing. The model formula used was:


adonis(distance_matrix∼Group+Age)

Here, Group corresponds to the four regional origins (Maotai, Zunyi, Jinsha, and Xishui), and Age represents the two aging stages (New and Aged). The distance matrix was calculated based on the 16S microbiome data (Bray–Curtis dissimilarity). We initially also tested a model including the interaction term Group:Age. However, the interaction effect was not statistically significant (*P* > 0.05) and explained only a negligible proportion of variance (<1%). To maintain model parsimony and facilitate interpretation, we opted for the additive model without interaction, focusing on the main effects of Region and Aging. Key MAGs contributing to aging were identified based on absolute abundance differences, with species names extracted from taxonomic classifications, and statistical testing was assessed using the Wilcoxon rank-sum test (two-group comparison). *P*-values from the test were adjusted for multiple comparisons using the Benjamini–Hochberg procedure to control the false discovery rate (FDR). FDR-adjusted *P*-value < 0.05 was used for the differential MAG detection.

### Non-targeted LC-MS metabolomics analysis of Daqu

Metabolites were extracted from Daqu solid samples following a standard LC-MS-based non-targeted metabolomics workflow. Briefly, 50 mg of finely ground Daqu powder was placed into a 2 mL centrifuge tube with a 6 mm grinding bead and extracted with 400 μL methanol:water (4:1, vol/vol) containing 0.02 mg/mL L-2-chlorophenylalanine as internal standard. Samples were homogenized (−10°C, 50 Hz, 6 min), then followed by ultrasound at 40 kHz for 30 min at 5°C, and centrifuged (13,000 × *g*, 4°C, 15 min). The supernatant was collected for LC-MS/MS analysis. A pooled quality control (QC) sample was prepared by mixing equal aliquots of all extracts and injected at regular intervals throughout the run.

Chromatographic separation was performed on an ACQUITY UPLC HSS T3 column (100 mm × 2.1 mm i.d., 1.8 µm; Waters, Milford, USA) using a Thermo UHPLC system coupled to a Q Exactive HF-X mass spectrometer. The mobile phases consisted of 0.1% formic acid in water:acetonitrile (95:5, vol/vol) (A) and acetonitrile:isopropanol:water (47.5:47.5, vol/vol) (B), with a flow rate of 0.40 mL/min and column temperature of 40°C. Mass spectra were acquired in both positive and negative ESI modes under DDA mode over m/z 70–1,050.

Raw data were processed using Progenesis QI 2.3 (Nonlinear Dynamics, Waters, USA) for peak detection, alignment, and normalization. Features present in ≥80% of samples were retained, missing values were imputed with the minimum value, and QC features with RSD >30% were removed. Multivariate analyses (PCA and OPLS-DA) were performed using the R package ropls. Differential metabolites (DEM) were identified using Student’s t-test combined with OPLS-DA VIP scores, all differential analyses using Benjamini–Hochberg correction for multiple testing, DEMs were defined by VIP >1 and *P* <0.05, and mapped into their biochemical pathways through metabolic enrichment and pathway analysis based on database search (KEGG, http://www.genome.jp/kegg/).

## Data Availability

All data in the study are publicly available. These data can be found at NCBI SRA and under BioProject ID with PRJNA1366088.

## SUPPLEMENTAL MATERIAL

Fig. S1 spectrum.03775-25-s0001.pdf CAZyme families carried by MAGs significantly enriched in new and aged Daqu.

Fig. S2 spectrum.03775-25-s0002.pdf Heatmap of the top 50 differential metabolites in new and aged Daqu samples.

Supplemental tables spectrum.03775-25-s0003.xlsx Tables S1 to S7.

SUPPLEMENTAL FILE 1 spectrum.03775-25-s0004.docx Supplemental figure legends.

ASM does not own the copyrights to Supplemental Material that may be linked to, or accessed through, an article. The authors have granted ASM a non-exclusive, world-wide license to publish the Supplemental Material files. Please contact the corresponding author directly for reuse.
