# Supplementary figures and images for: Multi-omics characterization of new and aged Daqu reveals region-specific microbial succession and metabolic signatures in Maotai-flavor liquor fermentation
Source: Microbiol Spectr. 2026 May 18;14(7):e03775-25. doi: 10.1128/spectrum.03775-25 (PMC13340048; doi:10.1128/spectrum.03775-25)

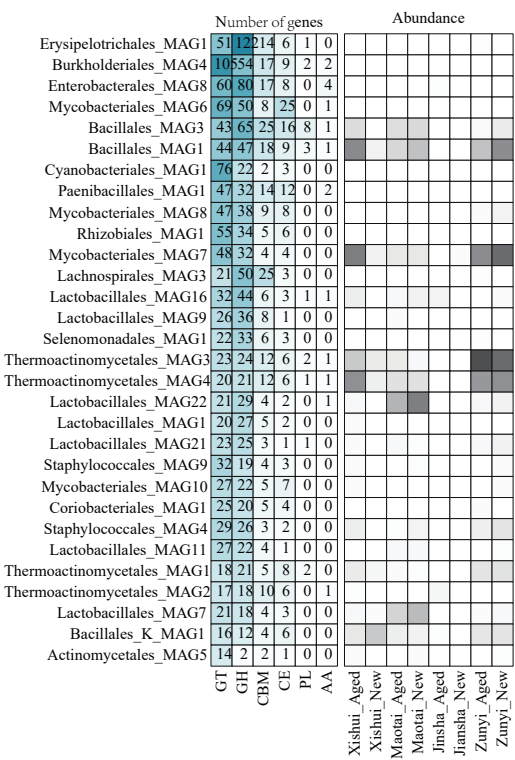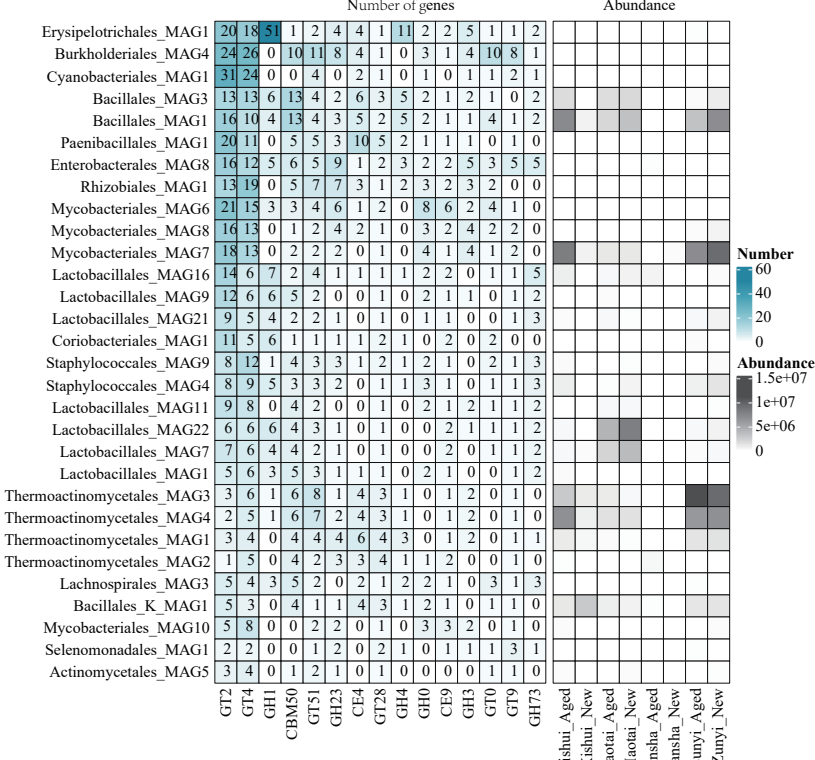

Supplement: Fig. S1 — CAZyme families carried by MAGs significantly enriched in new and aged Daqu. [file spectrum.03775-25-s0001.pdf]

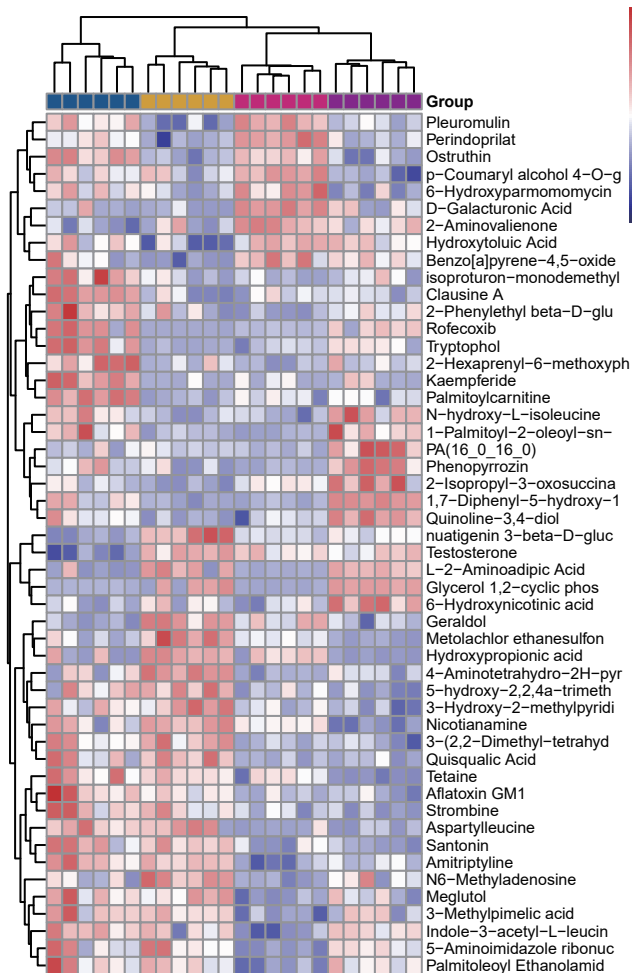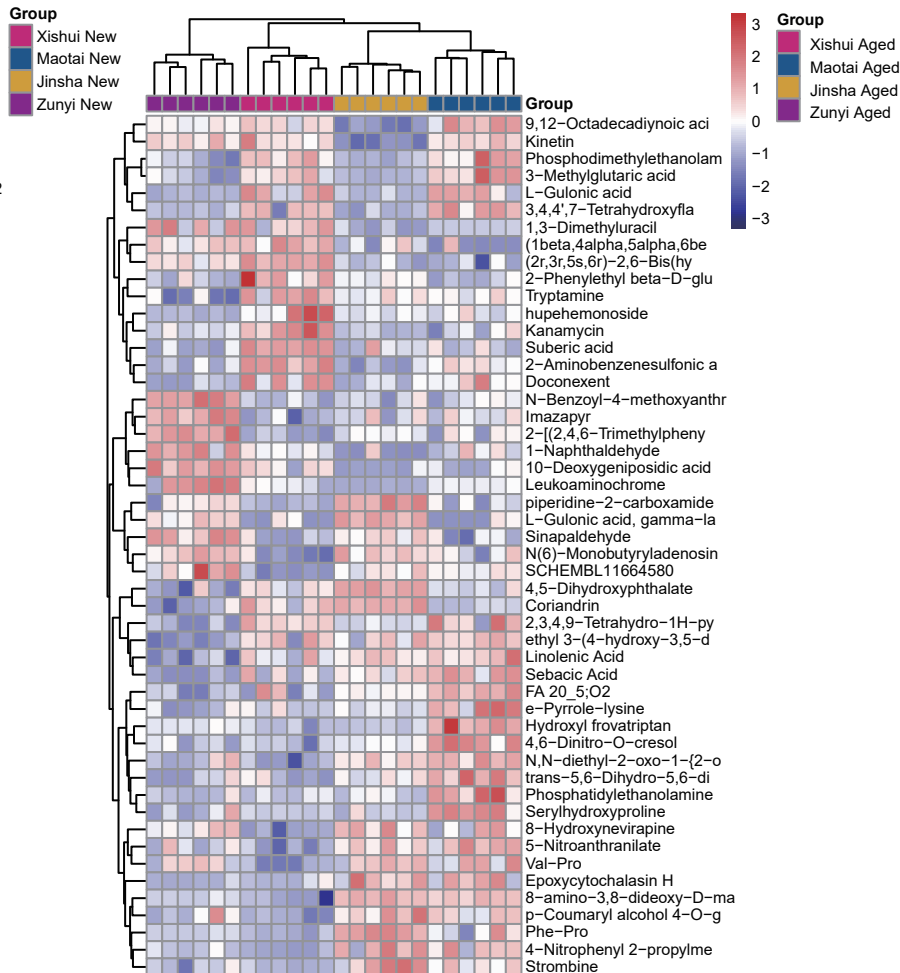

Supplement: Fig. S2 — Heatmap of the top 50 differential metabolites in new and aged Daqu samples. [file spectrum.03775-25-s0002.pdf]
