# Supplementary material for: Multi-omics characterization of new and aged Daqu reveals region-specific microbial succession and metabolic signatures in Maotai-flavor liquor fermentation
Source: Microbiol Spectr. 2026 May 18;14(7):e03775-25. doi: 10.1128/spectrum.03775-25 (PMC13340048; doi:10.1128/spectrum.03775-25)
Supplement: SUPPLEMENTAL FILE 1 — Supplemental figure legends. [file spectrum.03775-25-s0004.docx]

**Figure S1, CAZyme families carried by MAGs significantly enriched in new and aged Daqu.** (Left Panel) Comparison of the number of genes and relative abundance of specific microbial orders across the sampling sites. The heatmap displays the genomic potential for carbohydrate degradation within key taxa, including Erysipelotrichales, Burkholderiales, Bacillales, and Mycobacteriales. Colors represent the gene count (Number) and the total sequence abundance in each sample type. (Right Panel) Detailed profiling of specific CAZyme families (e.g., GH23, CE4, GT28, GH4, GH3, GT9) across the identified MAGs. Notable producers of extracellular enzymes, such as members of the Actinomycetota (Nocardiopsis meridipullorum, Saccharopolyspora rectivirgula, Streptomyces cacaoi, and Kroppenstedtia spp.), are shown to encode multiple CAZyme families, including GH43 and GH3, which are associated with the depolymerization of lignocellulose. The bar charts on the right summarize the total CAZyme gene numbers and overall microbial abundance across the Xishui, Maotai, Jinsha, and Zunyi regions, highlighting differences between "New" and "Aged" Daqu fermentation environments.

**Figure S2, Heatmap of the top 50 differential metabolites in new and aged Daqu samples.** The heatmap displays the relative abundance patterns of the top 50 significantly differential metabolites selected from a total of 653 identified differential features. Samples are grouped according to production stage (new vs. aged) and geographic origin (Xishui, Maotai, Jinsha, and Zunyi). Metabolite abundances were normalized and scaled prior to visualization, and are represented as Z-scores (color scale ranging from low [blue] to high [red] relative abundance). Distinct clustering patterns were observed between new and aged Daqu, with aged samples showing prominent enrichment of flavor-associated compounds, including ethyl esters and aromatic alcohols.
